# Histone Acetylation Accompanied with Promoter Sequences Displaying Differential Expression Profiles of B-Class MADS-Box Genes for *Phalaenopsis* Floral Morphogenesis

**DOI:** 10.1371/journal.pone.0106033

**Published:** 2014-12-11

**Authors:** Chia-Chi Hsu, Pei-Shan Wu, Tien-Chih Chen, Chun-Wei Yu, Wen-Chieh Tsai, Keqiang Wu, Wen-Luan Wu, Wen-Huei Chen, Hong-Hwa Chen

**Affiliations:** 1 Department of Life Sciences, National Cheng Kung University, Tainan, Taiwan; 2 Institute of Plant Biology, National Taiwan University, Taipei, Taiwan; 3 Institute of Tropic Plant Sciences, National Cheng Kung University, Tainan, Taiwan; 4 Orchid Research Center, National Cheng Kung University, Tainan, Taiwan; The National Orchid Conservation Center of China; The Orchid Conservation & Research Center of Shenzhen, China

## Abstract

Five B-class MADS-box genes, including four *APETALA3* (*AP3*)-like *PeMADS2*∼*5* and one *PISTILLATA* (*PI*)-like *PeMADS6*, specify the spectacular flower morphology in orchids. The *PI*-like *PeMADS6* ubiquitously expresses in all floral organs. The four *AP3*-like genes, resulted from two duplication events, express ubiquitously at floral primordia and early floral organ stages, but show distinct expression profiles at late floral organ primordia and floral bud stages. Here, we isolated the upstream sequences of *PeMADS2∼6* and studied the regulatory mechanism for their distinct gene expression. Phylogenetic footprinting analysis of the 1.3-kb upstream sequences of *AP3*-like *PeMADS2*∼*5* showed that their promoter regions have sufficiently diverged and contributed to their subfunctionalization. The amplified promoter sequences of *PeMADS2*∼*6* could drive *beta-glucuronidase* (GUS) gene expression in all floral organs, similar to their expression at the floral primordia stage. The promoter sequence of *PeMADS4*, exclusively expressed in lip and column, showed a 1.6∼3-fold higher expression in lip/column than in sepal/petal. Furthermore, we noted a 4.9-fold increase in histone acetylation (H3K9K14ac) in the translation start region of *PeMADS4* in lip as compared in petal. All these results suggest that the regulation via the upstream sequences and increased H3K9K14ac level may act synergistically to display distinct expression profiles of the *AP3*-like genes at late floral organ primordia stage for *Phalaenopsis* floral morphogenesis.

## Introduction

In *Arabidopsis thaliana* and *Antirrhinum majus*, the development of different floral organs is controlled by several classes of floral-organ identity genes [1]. All these genes, except *AP2* from *A. thaliana*, belong to the MADS-box family, with a highly conserved 180-bp sequence of the MADS domain that can bind to the conserved CArG-box [CC(A/T)_6_GG] sequence [2], [3]. These MADS-box genes were cloned from a wide range of plant species to explain the floral organ development [1], [4]–[6]. The diversification of MADS-box genes during evolution has been proposed to be a major driving force for floral diversity in land plant architecture [1], [7].

Various regulatory strategies have been reported for the expression of MADS-box genes in *Arabidopsis*, including transcriptional regulation on the upstream sequences or intron regions by transcription factors, with feedback and feed-forward loops, and epigenetic regulation by small RNAs [8]. Discrete *cis*-acting elements on the B-class *APETALA3* (*AP3*) and *PISTILLATA* (*PI*) promoters are responsible for their expression in petal and stamen [9]–[11]. In addition, the first and second introns of *FLOWERING LOCUS C* (*FLC*) [12] and *AGAMOUS* (*AG*) [13], respectively, have a role in regulating spatial or temporal gene expression patterns. For epigenetic control of gene expression, both dimethyl histone H3 lysine-9 (H3K9me2) and dimethyl histone H3 lysine-27 (H3K27me2) are the gene repression markers. In contrast, both trimethyl histone H3 lysine-4 (H3K4me3) and acetyl histone H3 (H3Ac) are the active histone markers.

Orchidaceae is one of the largest families of flowering plants. The high species diversity in orchids is largely due to their adaptation to specialized insect pollination [14]. The orchid flower is spectacular with a gynostemium or column (a fusion of the male and female reproductive organs) and a highly modified petal, the labellum or lip, which offers a landing platform for pollinators [14]–[16].

In *Phalaenopsis* orchids, four *AP3*-like and one *PI*-like B-class MADS-box genes, *PeMADS2*∼*6*, have been isolated and characterized for their roles in flower morphogenesis [17]–[19]. Two duplication events resulted in the four *AP3-*like *PeMADS2*∼*5*. The first, occurring early in the evolutionary history of Orchidaceae, resulted in *AP3A* and *AP3B* clades, and the second resulted in four subclades, *AP3A1* (*PeMADS3*), *AP3A2* (*PeMADS4*), *AP3B1* (*PeMADS2*), and *AP3B2* (*PeMADS5*) [20]–[22]. Fluorescence *in situ* hybridization revealed that the four *AP3*-like *PeMADS2*∼*5* genes are located on different chromosomes of *P. equestris*, so the four orchid *AP3* paralogs may have been resulted from genome duplication [20]. The effects of gene duplication and their differences on gene regulation are important in the diversity and evolution of flowering plants [23]–[25]. At the floral primordia and early floral organ primordia stages, the transcripts of *PeMADS2*∼*5* were detected ubiquitously, and then they are constrained to distinctively expressed organs at the late floral organ primordia stage and floral bud stage: *PeMADS2* mainly expresses in sepal and petal, *PeMADS3* predominantly expresses in petal and lip, *PeMADS4* exclusively expresses in lip and column, and *PeMADS5* is mainly expressed in petal [17], [20]. In contrast, the *PI*-like *PeMADS6* is ubiquitously expressed in sepal, petal, lip, and column [18]. The ‘Orchid code’ assumes that the differential expression of B-class genes determined the development of sepal, petal, lip, and column [26], [27]. Moreover, the ‘homeotic orchid tepal’ (HOT) model are proposed for the dualistic features of duplicated B-class MADS-box genes involved in orchid perianth development and growth [20].

Although the five B-class MADS-box genes play important roles in the perianth development in orchids, the regulatory strategies for their distinct expression profiles in various floral organs have not been characterized. In this study, we identified the upstream promoter sequences of *PeMADS2*∼*6* in *Phalaenopsis* orchids and used phylogenetic footprinting to identify conserved motifs among these promoter sequences. We analyzed the promoter activity of the upstream sequences of *PeMADS2*∼*6* for driving GUS and luciferase gene expression in various floral organs. In addition, we examined the regulatory effects of the intron region, DNA methylation, and histone modification for their association with the high expression level of *PeMADS4* in lip.

## Materials and Methods

### Plant materials

All upstream sequences of *PeMADS* genes were isolated from *P. equestris* with red sepal, petal and orange lip [17]. *P. aphrodite* subsp. *formosana* with white sepal, petal and yellow lip was purchased from Taiwan Sugar Corp. (Tainan, Taiwan) and used in particle bombardment experiments because the white sepal and petal made it easier for GUS staining. All plant materials were grown in the greenhouse at National Cheng Kung University (Tainan, Taiwan) under natural light and controlled temperature from 23°C to 27°C.

### Isolation of the upstream promoter sequences of *PeMADS2∼6*

Genomic DNA was extracted from young flower buds by the cetyltriammonium bromide (CTAB) method [28]. The upstream promoter sequences of *PeMADS2*∼*6* were isolated by use of the Universal GenomeWalker Kit (Clontech, Palo Alto, CA, USA). The desired DNA fragment was obtained by two successive PCR-based rounds of screening the GenomeWalker libraries and checked by agarose gel electrophoresis. The major bands were recovered from gels with use of the Gel DNA Fragment Extraction Kit (Geneaid, New Taipei City, Taiwan), and cloned into the pGEM-T Easy Vector (Promega, Madison, WI, USA). We randomly selected 10 to 12 colonies for sequencing. The promoter sequences were compared to all known DNA sequences with use of the default settings of BLASTN from NCBI (www.ncbi.nlm.nih.gov).

When genome walking could not extend the upstream regulatory sequences for *PeMADS3∼5* genes, we used BAC clones constructed from *P. equestris* [29] for promoter identification. Southern blot hybridization was used to identify BAC clones containing various *PeMADS* genes with the gene-specific probes used in previous study [17], [18]. The BAC DNAs of each clone were isolated following the standard method [30], then digested with *Hin*dIII and separated by electrophoresis in 0.7% agarose gel. The resolved bands corresponding to promoter sequences with hybridized signals were recovered with use of the Gel DNA Fragment Extraction Kit (Geneaid) and cloned into the pGEM-T Easy vector (Promega). We randomly selected 10 to 12 colonies for sequencing. The promoter sequences characterized are deposited at the NCBI site under the accession numbers: *PeMADS2* promoter (KJ127932), *PeMADS3* promoter (KJ127933), *PeMADS4* promoter (KJ127931), *PeMADS5* promoter (KJ127934), and *PeMADS6* promoter (KJ127935).

### Promoter sequence analysis

We used the PLACE software (A database of Plant *cis*-acting Regulatory DNA Elements, http://www.dna.affrc.go.jp/PLACE/index.html) to predict the putative CArG box having 10-nt matches with the C(A/T)_8_G sequence [31]. In addition, the CArG box sequence was predicted for a standard of 9 of 10 matches with the core consensus binding site CC(A/T)_6_GG [9] by using a homemade software (designed by Dr. Chih-Hsiung Fu, Department of Engineering Science, National Cheng Kung University).

Since only 1.3-kb upstream regulatory sequence of *PeMADS3* was cloned, we then chose the 1.3-kb upstream sequences of *PeMADS2∼5* for phylogenetic footprinting analysis by FOOTPRINTER [32]. This tool takes into account the evolutionary relationships and the phylogenetic tree analysis. The prediction of a conserved 10-bp or 11-bp motif with a 0-bp mutation allowance was performed and the motif losses were allowed to identify the conserved motif within two promoter sequences. For all other parameters, default values were used.

### Construction of chimeric reporter gene fusions

All the promoter sequences of *PeMADS2*∼*6* were amplified directly from the genomic DNA of *P. equestris* by using PCR with the length of 3,249, 1,293, 3,303, 2,062, and 1,514 bp, respectively, and constructed in-frame translational fusions with the GUS reporter gene in pBI221 vector. Serial deletion fragments of *PeMADS2∼6* promoters were also cloned by PCR-amplification with a series of forward primers and a reverse primer (S1 Table). The resulting PCR products were cloned into pGEM-T Easy vector (Promega) and then digested with *Sph*I and *Bam*HI. pBI221 containing the GUS reporter gene was digested with the same enzymes to replace the cauliflower mosaic virus (CaMV) 35S promoter with the serial deletion fragments of promoter sequences. All constructs were confirmed by sequencing to eliminate possible PCR-introduced mutations. Both the pBI221 vector containing the CaMV 35S promoter-GUS fusion (pBI221) and pBI221 vector containing a promoterless GUS cassette (pBI-PL) were recruited and considered as positive and negative controls, respectively.

### Transient transformation by particle bombardment

The promoter deletion-GUS fusion plasmids were isolated by use of the High-Speed Plasmid Mini kit (Geneaid) and coated on gold particles 1.6* µ*m in diameter by coprecipitation as described [33]. Before particle bombardment, each floral organ was separated from the floral buds and placed on a central core 2 cm in diameter on solid agar medium. Promoter constructs were bombarded into various floral organs by use of Model Biolistic PDS-1000/He (BioRad, Hercules, CA, USA) at 1,100 psi helium gas pressure, 28.5-inch Hg vacuum and 9-cm target distance. After bombardment, floral organs were incubated at 23°C to 27°C for 2 days in an incubator with a 10-h/14-h light-dark photoperiod until analyzed by GUS histochemical staining and quantitative dual luciferase assays.

### GUS histochemical staining assay

Histochemical staining of GUS activity was performed as described [34]. Tissues for GUS staining were vacuum-infiltrated in GUS assay buffer (1 mg/ml 5-bromo-4-chloro-3-indoyl glucuronide [X-Gluc]; 50 mM sodium phosphate, pH 7.0; 10 mM EDTA, pH 8.0; 0.5 mM potassium ferricyanide, 0.5 mM potassium ferrocyanide and 0.1% Triton X-100) and incubated at 37°C overnight. Stained tissues were cleared of chlorophyll in 70% ethanol and then photographed under a microscope (TMS-F, Nikon). For each promoter-GUS fusion, the GUS staining pattern was analyzed in four independent bombarded buds, and repeated three times independently.

### Quantitative dual luciferase assay

Serial-deleted promoter fragments were obtained by digestion of *PeMADS4* and *PeMADS6* promoters in pBI221 with *Sph*I and *Bam*HI, and then ligated into pJD301, containing a firefly (*Photinus pyralis*) luciferase gene, to replace the CaMV 35S promoter for the serial pJD-Pe4p and pJD-Pe6p constructs. pJD301_R, with *Renilla* luciferase gene driven by the CaMV 35S promoter, was an internal control to normalize transfection efficiency.

On day 2 after bombardment, each sample was ground, and then 1 X Passive Luciferase Buffer (Promega, Madison, WI, USA) was added. Luciferase activity was measured by use of the dual-luciferase reporter assay system (Promega) with a Lumat LB 9507 Luminometer (Berthold Technologies, Bad Wildbad, Germany), a 10-sec pre-measurement delay and a 10-sec measurement period for each assay. The relative luciferase activity was calculated as the ratio of firefly to *Renilla* luciferase activity. For each analysis, two independent buds were bombarded and analyzed, and the bombardments were repeated three times independently. Statistical analysis was performed by T-test, and the differences were considered significant at p<0.01.

### Southern blot hybridization

Genomic DNA isolated from sepals, petals, lips, and columns of *P. equestris* was digested with restriction enzymes *Hpa*II and *Msp*I or *Dra*I and *Hpa*II, resolved on 0.8% agarose gel, and transferred to nylon filters (Amersham Pharmacia Biotech, Piscataway, NJ, USA) by use of a vacuum transfer system (Amersham Pharmacia Biotech). The recognition sites of *Hpa*II and *Msp*I are both CCGG, but digestion of *Hpa*II is blocked by cytosine methylation and *Msp*I is blocked by only cytosine methylation within the first cytosine (CpCGG). Two probes of *PeMADS4* for methylation Southern blot assay were used: probe 1 contained the promoter and 5′ UTR sequences, and probe 2 contained the 5^th^ intron regions. The primers were listed in S1 Table. Southern blot hybridization was performed and followed the standard protocol [30] with the ^32^P-labeled probes prepared by a PCR strategy.

### Bisulfite sequencing

Bisulfite sequencing analysis was carried out with the EpiTect Bisulfite kit (QIAGEN, Hilden, Germany). 2-*µ*g genomic DNAs from the petal and lip of *P. equestris* were treated with conversion reagents and then cleaned up as described in the manufacturer's instruction. The DNA was served as a template for PCR amplification with the incubation at 94°C for 5 min, thermocycling for 35 cycles (94°C for 30 s, 55°C for 30 s, and 72°C for 1 min), and finally at 72°C for 7 min with the primers listed in S1 Table. The PCR products were cloned into pGEM-T Easy vector (Promega) and transformed into *Escherichia coli*. We randomly selected 10 colonies for sequencing and analysis.

### Chromatin immunoprecipitation (ChIP) and real-time PCR analyses

ChIP assay was as described [35]. Chromatin extracts were prepared from the petal and lip of *P. equestris* treated with formaldehyde. Chromatin was sheared to an average length of 500–1500 bp by sonication and immunoprecipitated with the antibodies anti-H3K4me3 (catalogue no. 04-745, Millipore, Billerica, MA, USA), anti-H3K9me2 (catalogue no. 04-768), or anti-H3K9K14ac (catalogue no. 06-599). The immunocomplexes were harvested with Protein G agarose beads (Millipore) and heated at 65°C for 5 hours to release DNA cross-linked to the immunoprecipitated proteins. The DNA cross-linked to the immunoprecipitated proteins was analyzed by real-time PCR with the primers listed in S1 Table. The immunoprecipitations were performed twice.

The DNA template was mixed with 2X SYBR Green PCR master mix (Applied Biosystems, Foster City, CA, USA) in an ABI Prism 7000 sequence detection system (Applied Biosystems), and each sample was analyzed in triplicate. Reactions involved incubation at 95°C for 10 min, and thermocycling for 40 cycles (95°C for 15 s and 60°C for 1 min). After amplification, melting curve analysis was used to verify amplicon specificity and primer dimer formation. The amount of DNA after ChIP was quantified and normalized to an internal control *ACTIN2* for H3K4me3 and H3K9K14ac or *Ta3* for H3K9me2 [36]. Data are mean ± SD calculated from three technological and two biological replicates.

## Results

### Cloning of the upstream sequences of *PeMADS2∼6*

**Figure 1:**
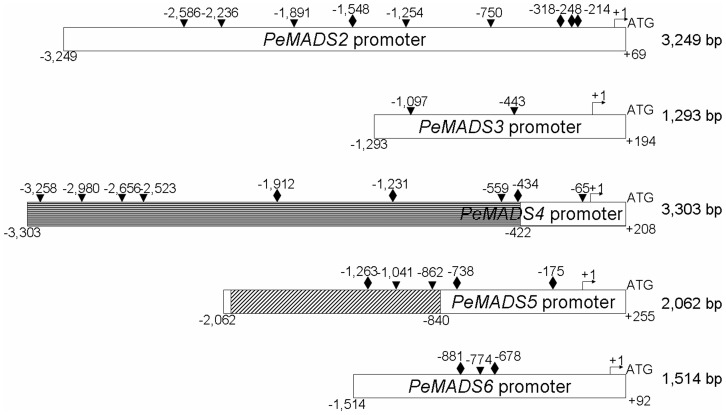
Promoter sequences of *PeMADS2∼6* and the putative CArG boxes. Length of promoter sequences of *PeMADS2, PeMADS3*, and *PeMADS6* were 3,249, 1,293 and 1,514 bp, respectively. The promoter sequence of the *PeMADS4* was extended from −422 bp to −3,303 bp (horizontal line box), and the original fragment of −2,121 to −840 bp of *PeMADS5* promoter was replaced by a 1,227-bp fragment (diagonal line box). The rhombus and the triangles indicate the putative CArG boxes predicted by consensus CC(A/T)_6_GG and C(A/T)_8_G motifs, respectively. “+1” means the transcription start site and ATG was the translation start site. The lengths of the promoters are show from the transcription start site to the upstream sequences.

The five *PeMADS* promoter regions were cloned from genomic DNA by use of GenomeWalker. The *PeMADS2*∼*6* promoter fragments obtained were 3,249, 1,293, 422, 2,121, and 1,514 bp, respectively (Fig. 1). However, the 2,121-bp promoter sequence of *PeMADS5* could not be amplified from the genomic DNA of *P. equestris* by using PCR, and both *PeMADS3* and *PeMADS4* with the promoter fragments <1.5 kb could not be extended. We then used BAC clones of *P. equestris* for promoter identification of *PeMADS3∼5* by Southern blot hybridization with probes from the coding sequences of *PeMADS3*∼*5* (Table 1). BAC DNA was isolated and confirmed by restriction enzyme digestion. The resolved bands corresponding to the hybridized signals were recovered and sequenced. After assembly, the *PeMADS3* promoter remained at 1,293 bp. For the *PeMADS4* promoter, a 4.8-kb DNA fragment from BAC clones was recovered and sequenced, which extended its upstream sequence to 3,303 bp (Fig. 1, horizontal line box). For *PeMADS5*, the promoter fragment was replaced with a 2,062-bp fragment (Fig. 1).

**Table 1:** BAC clones containing various *PeMADS* genes.

Genes	Clone no.	BAC clones
*PeMADS3*	3	NCKU-PE-btBAC-2050 C15
		NCKU-PE-btBAC-2065 P3
		NCKU-PE-btBAC-3081 A24
*PeMADS4*	1	NCKU-PE-btBAC-1105 H24
*PeMADS5*	4	NCKU-PE-btBAC-2016 E10
		NCKU-PE-btBAC-2022 D13
		NCKU-PE-btBAC-2035 M21
		NCKU-PE-btBAC-2049 A22

### Prediction of CArG box in the promoter sequences of *PeMADS2∼6*

We used an in-house developed software to predict the presence of the putative CArG box with a standard of a 9-in-10-nt match with the core consensus CC(A/T)_6_GG motif [9]. Four CArG boxes were detected at nucleotides −1,548, −318, −248, and −214 in the *PeMADS2* promoter region; three at nucleotides −1,912, −1,231, and −434 in the *PeMADS4* promoter region; three at nucleotides −1,263, −738, and −175 in the *PeMADS5* promoter region, and two at nucleotides −881 and −678 in the *PeMADS6* promoter region (Fig. 1, Table 2). In contrast, no CArG-box-like sequence was detected in the *PeMADS3* promoter sequence.

**Table 2:** Putative CArG boxes at the *PeMADS* promoter regions predicted by homemade software and the PLACE database.

*PeMADS* promoter (bp)	CArG-box sequences	Location (nt at the promoter regions)
*PeMADS2*	CCCTAAATGG	−214^a^
(3,249)	CCATTCTAGG	−248^a^
	CTTTAAATGG	−318^a^
	CTATATTAAG	−750^b^
	CATAATTTTG	−1,254^b^
	CCAAAATTTG	−1,548^a^
	CTAATTTTAG	−1,891^b^
	CAAAATTTAG	−2,236^b^
	CATATTAAAG	−2,586^b^
*PeMADS3*	CAAAAAAAAG	−443^b^
(1,293)	CTTTTATAAG	−1,097^b^
*PeMADS4*	CTTATAAAAG	−65^b^
(3,303)	CTATTATAGG	−434^a^
	CATATTATAG	−559^b^
	CATATTTTGG	−1,231^a^
	CCTATGTAGG	−1,9128^a^
	CATATATTAG	−2,523^b^
	CTTTTTTATG	−2,656^b^
	CAAAATTTTG	−2,980^b^
	CAAAATTTTG	−3258^b^
*PeMADS5*	GCTTAATTGG	−175^a^
(2,062)	TCAAAATTGG	−738^a^
	CATAAATATG	−862^b^
	CTTTATATTG	−1,041^b^
	CGATTTAAGG	−1,263^a^
*PeMADS6*	CCAAATTTGA	−678^a^
(1,514)	CAAATTTAAG	−774^b^
	GCAAAATAGG	−881^a^

a CArG boxes predicted with a homemade software.

b CArG boxes predicted with the PLACE database.

We used the PLACE database with a standard of 10-nt match with the C(A/T)_8_G sequence to further examine the CArG box [31]. Five CArG boxes were detected at nucleotides −2,586, −2,236, −1,891, −1,254, and −750 in the *PeMADS2* promoter region; two at nucleotides −1,097 and −443 in the *PeMADS3* promoter region; six at nucleotides −3,258, −2,980, −2,656, −2,523, −559, and −65 in the *PeMADS4* promoter region; and two at nucleotides −1,041 and −862 of the *PeMADS5* promoter region; one at nucleotide −774 in the *PeMADS6* promoter region (Fig. 1, Table 2).

### The conserved regulatory motifs predicted within the promoter regions of *PeMADS2∼5*

**Figure 2:**
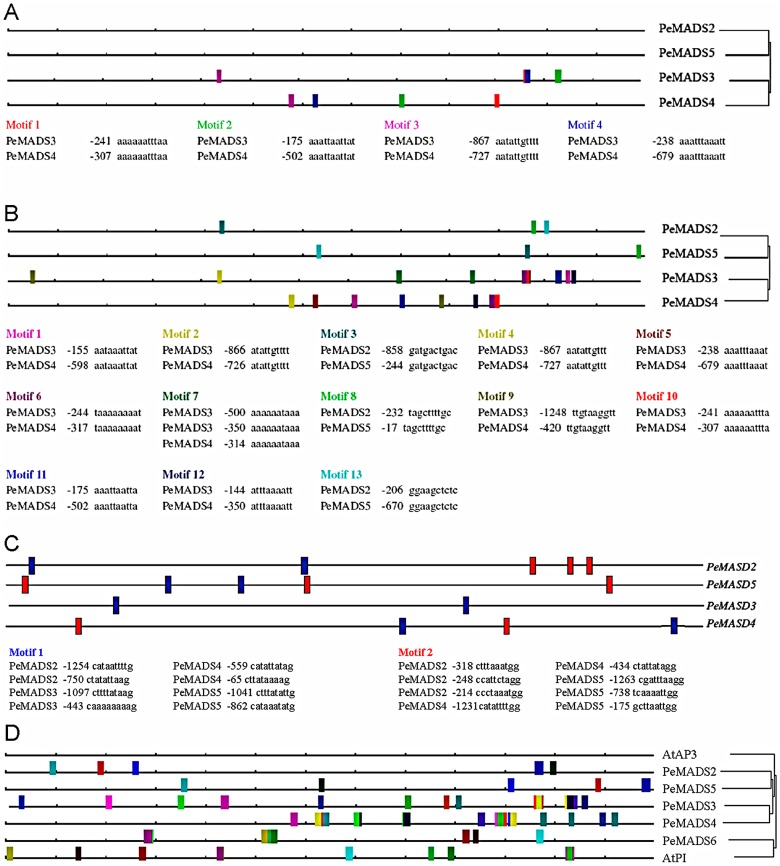
Visual representation of motifs in the 1.3-kb promoter sequences of *PeMADS2∼5*. FOOTPRINTER parameters: (A) motif size: 11, allowed mutations: 0, (B) motif size: 10, allowed mutations: 0. (C) Putative CArG boxes in the *PeMADS* promoter regions predicted with the homemade software (red box) and the PLACE database (blue box).

The conserved regulatory motifs are thought to be functional important for gene expression profiles [37]–[40]. However, multiple alignment of these promoter sequences using global alignment procedures was failed because the inversions often cause rearrangements of the regulatory elements [41]. Therefore, we examined the 1.3-kb upstream sequences of *PeMADS2*∼*5* by phylogenetic footprinting, a method for discovering regulatory elements in a set of regulatory regions [32] and has been used to promoter analysis for MADS-box genes in *Arabidopsis* and *Orchis italica* and for bZIP genes in rice and sorghum [40], [42], [43]. With prediction of a conserved 11-bp motif with a 0-bp mutation allowance, conservation of four 11-bp motifs was identified between the promoter regions of *PeMADS3* and *PeMADS4* in different order (Fig. 2A), while no motifs were conserved between promoter regions of *PeMADS2* and *PeMADS5* (Fig. 2A). With prediction of conserved 10-bp motifs, increased conserved motifs were identified between the promoter regions of *PeMADS3* and *PeMADS4*, and three motifs were lined up in those of *PeMADS2* and *PeMADS5* (Fig. 2B). Interestingly, we noticed that differential conserved 10-bp motif sets were detected between the promoter regions of *PeMADS2*/*PeMADS5* and *PeMADS3*/*PeMADS4* (Fig. 2B), which suggests that the two lineage of *PeMADS2/5* and *PeMADS3/4* have diverged for their subfuncationalization after gene duplication. Moreover, the CArG boxes were broadly distributed in the promoter regions of the four *PeMADS* genes, and no clear correlations of these CArG boxes were detected with their distinct expression profiles (Fig. 2C). Furthermore, with prediction of a conserved 12-bp motif with a 1-bp mutation allowance, motifs were identified between the promoter regions of *PeMADS2* and *PeMADS5*, *PeMADS3* and *PeMADS4*, and *PeMADS6* and *AtPI*, but no motifs were present between the promoter sequences of *AtAP3* and *PeMADS2∼5* (Fig. 2D).

### Functional analysis of *PeMADS2∼6* promoter sequences

**Figure 3:**
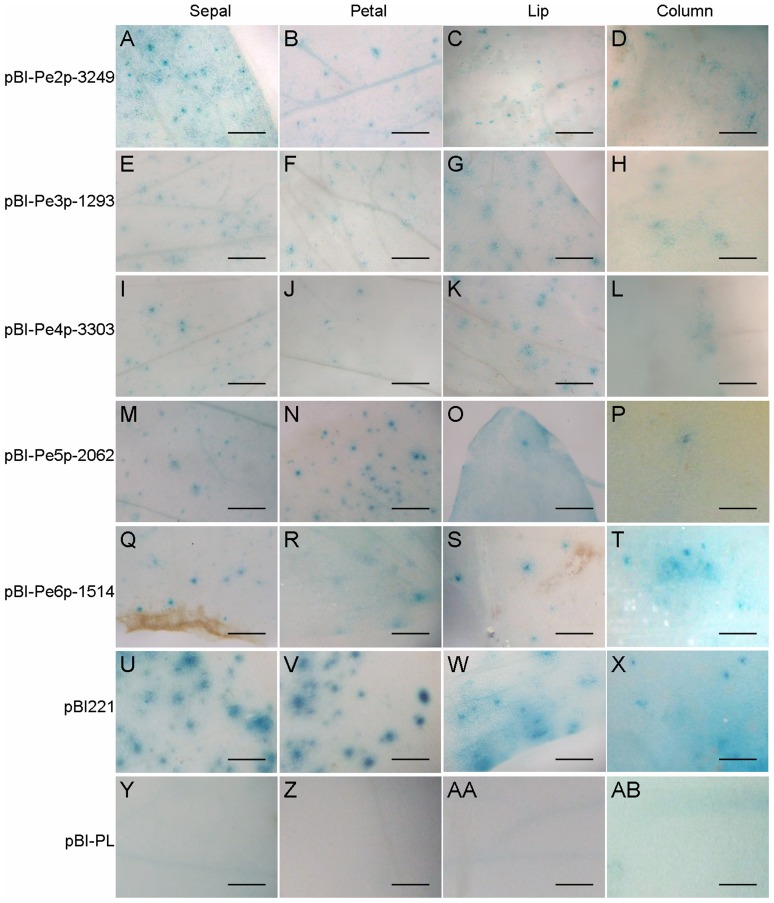
GUS histochemical staining for the promoter activities of *PeMADS2*∼*6*. Histochemical assay of GUS expression in floral organs shown in the order of pBI-Pe2p-3249 (A–D), pBI-Pe3p-1293 (E–H), pBI-Pe4p-3303 (I–L), pBI-Pe5p-2062 (M–P), pBI-Pe6p-1514 (Q–T), pBI221 (U–X) and pBI-PL (Y-AB). Constructs were bombarded into four independent floral buds, and results are representative of three independent bombardment experiments. Scale bar  =  0.5 mm.

We examined the promoter activities of *PeMADS2*∼*6* fragments with length of 3,249 bp, 1,293 bp, 3,303 bp, 2,062 bp, and 1,514 bp, respectively, for their ability to drive GUS expression by bombarding them into 1.5-cm floral buds of *P. aphrodite* subsp. *formosana*. Notably, all five PCR-amplified promoter fragments could drive GUS expression in the floral organs examined (Fig. 3A–T), similar to their expression patterns at the early floral primordia stage [20]. Moreover, serial deletion clones of the upstream sequences of *PeMADS2∼6* were constructed for GUS expression assay.

### The 208- and 375-bp promoter sequences of *PeMADS6* and *PeMADS4*, respectively, were sufficient to drive GUS expression

**Figure 4:**
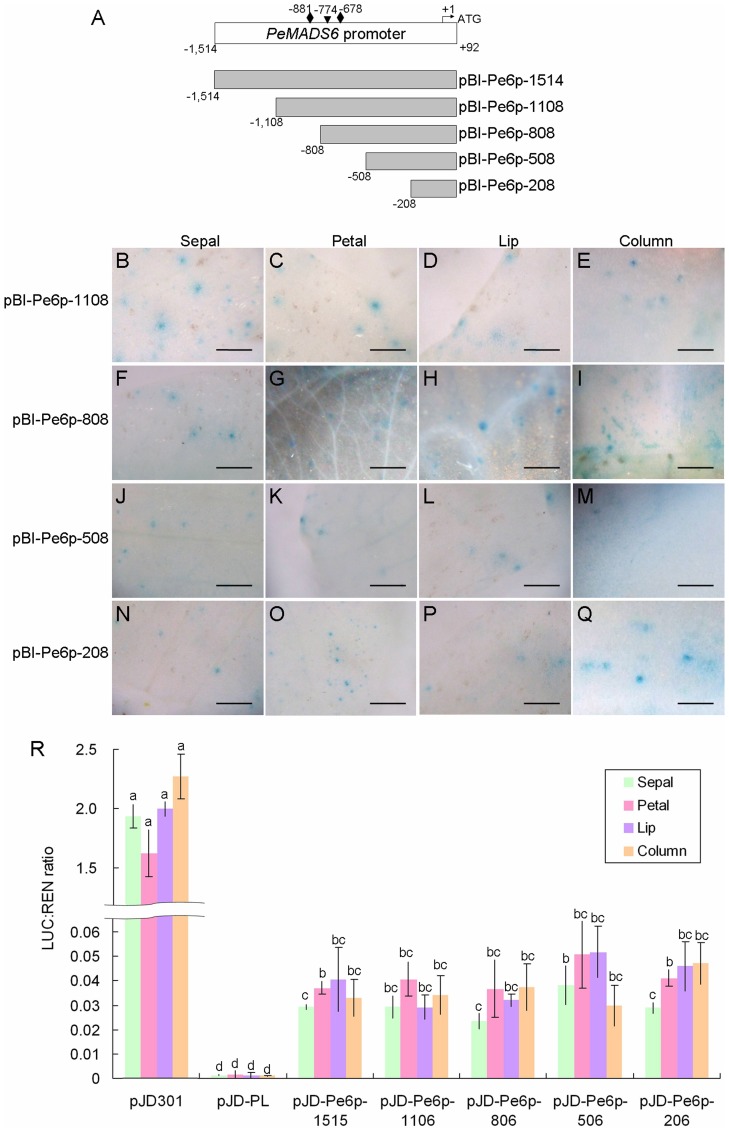
Functional analysis of serial deletions of *PeMADS6* promoter. (A) Serial deletion constructs of *PeMADS6* promoter. (B-Q) Histochemical assay of flower organs bombarded with serial deletions of *PeMADS6* promoter shown in the order of pBI-Pe6p-1108 (B–E), pBI-Pe6p-808 (F–I), pBI-Pe6p-508 (J–M) and pBI-Pe6p-208 (N–Q). Constructs were bombarded into four independent floral buds, and results are representative of three independent bombardment experiments. Scale bar  = 0.5 mm. (R) Dual luciferase assay of serial deletions of *PeMADS6* promoter. The same letters above the bars are not statistically different by T-test analysis (p<0.01). Data are mean ± SD (*n* = 6). All constructs were analyzed for promoter activities of driving luciferase expression by bombardment into two floral buds, and results are representative of three independent bombardment experiments.

The *PI*-like *PeMADS6* was expressed ubiquitously in all floral organs. To assess the minimal promoter region of *PeMADS6*, four deletion clones of the *PeMADS6* promoter sequence were resulted, including 1,108-bp, 808-bp, 508-bp, and 208-bp fragments containing 3, 2, 0, and 0 CArG boxes, respectively. Similar to the full length *PeMADS6* promoter construct, pBI-Pe6p-1514 (Fig. 3Q-T), all four deletion promoter sequences of *PeMADS6* could drive GUS expression in all the floral organs examined (Fig. 4B–Q), although the expression was slightly decreased in pBI-Pe6p-508 and pBI-Pe6p-208 constructs (Fig. 4J–Q). Moreover, quantitative dual luciferase assay was performed to further examine the differential promoter activities of these serial deletion constructs. The pJD-Pe6p-208 construct was sufficient to drive luciferase expression in all floral organs (Fig. 4R). Extension of the upstream sequence from the pJD-Pe6p-508 to pJD-Pe6p-1514 constructs conferred similar GUS expression in all floral organs (Fig. 4R). Therefore, the 208-bp promoter sequence was a minimal promoter for *PeMADS6* expression in all four floral organs.

**Figure 5:**
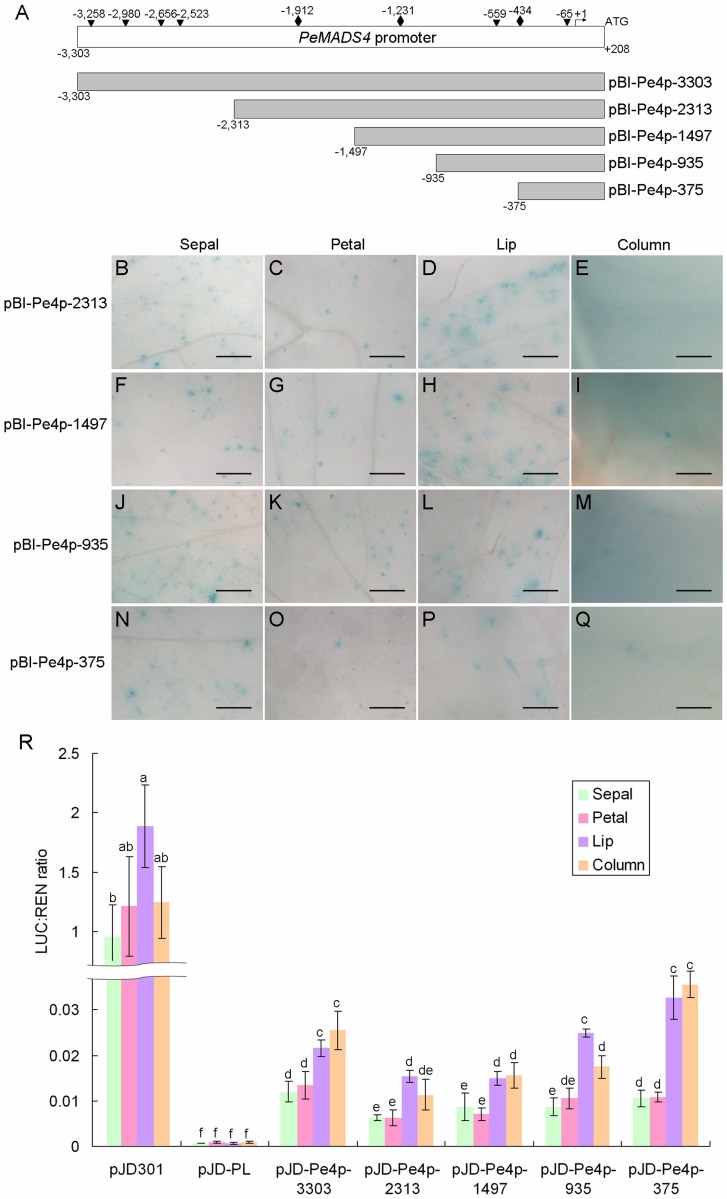
Functional analysis of serial deletions of *PeMADS4* promoter. (A) Serial deletion constructs of *PeMADS4* promoter. (B–Q) Histochemical assay of flower organs bombarded with serial deletions of *PeMADS4* promoter shown in the order of pBI-Pe4p-2313 (B–E), pBI-Pe4p-1497 (F–I), pBI-Pe4p-935 (J–M) and pBI-Pe4p-375 (N–Q). Constructs were bombarded into four independent floral buds, and results are representative of three independent bombardment experiments. Scale bar  = 0.5 mm. (R) Dual luciferase assay of the serial deletions of *PeMADS4* promoter. The same letters above the bars are not statistically different by T-test analysis (p<0.01). Data are mean ± SD (*n* = 6). All constructs were analyzed for promoter activities of driving luciferase expression by bombardment into two floral buds, and results are representative of three independent bombardment experiments.

The four *AP3*-like *PeMADS2∼5* genes express differentially in the floral organs with distinct patterns [17]. Two to five serial deletion clones for the upstream regulatory sequences of *PeMADS2∼5* were constructed for GUS expression assay. Similarly, most serial deletion constructs could drive GUS expression in all four floral organs (Fig. 5B–Q, S1–S3 Figures), resembled to the gene expression patterns at the early floral organ primordia stage. Among them, the minimal promoters of *PeMADS2∼5* were found to be 291 bp, 407 bp, 375 bp, and 122 bp of their upstream regulatory sequences, respectively (Fig. 5N–Q, S1–S3 Figures).

The promoter sequence of *PeMADS4* was chosen for further examination by using quantitative dual luciferase assay to delineate its exclusive expression in lip and column at the late floral primordia stage [20]. All five serial deletion promoter constructs of *PeMADS4* with various lengths conferred a 1.6∼3-fold increase of luciferase activities in lip and column than in sepal and petal (Fig. 5R), in accordance with the high expression of *PeMADS4* in lip and column. The shortest fragment, pJD-Pe4p-375 conferred a 3-fold higher promoter activity in lip and column than in sepal and petal (Fig. 5R), which suggests that the upstream sequences of *PeMADS4* was necessary, but not sufficient for its exclusive high expression in lip and column. It is possible that other factors are also required for regulating the lip- and column-specific expression of *PeMADS4* at the late floral organ primordia stage of *Phalaenopsis* orchids.

### The 5^th^ intron of *PeMADS4* had no effect on its organ-specific expression pattern

**Figure 6:**
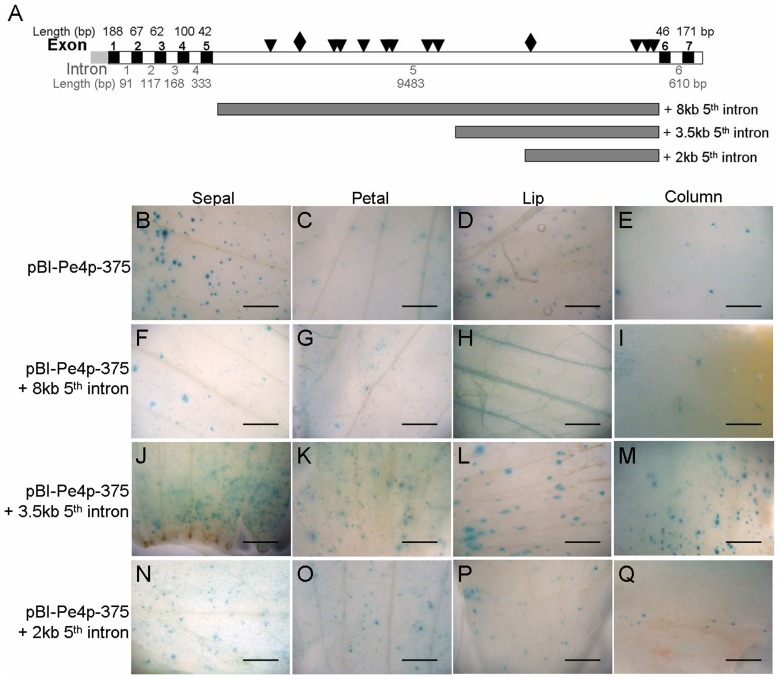
Histochemical assay of the 5^th^ intron of *PeMADS4*. (A) Genomic structure of *PeMADS4*. Gray, black, and white boxes indicate the promoter, exon, and intron regions of *PeMADS4* gene, respectively. Numbers above the black boxes are the number and length (bp) of exons, respectively. Numbers beneath the white boxes are the number and length (bp) of introns, respectively. Two CC(A/T)_6_GG sequences (rhombus) and 11 C(A/T)_8_G sequences (triangles) are located in the 5^th^ intron. Three serial deletions of the 5^th^ intron were designed for 2-, 3.5- and 8-kb sequences, respectively, and inserted into the upstream region of the Pe4pF1 promoter sequence in the pBI-Pe4p-375 construct. (B-Q) Histochemical assay of the serial deletions of the 5^th^ intron of *PeMADS4* were in the order of pBI-Pe4p-375 (B–E), pBI-Pe4p-375-8- (F–I), pBI-Pe4p-375-3.5- (J–M) and pBI-Pe4p-375-2-kb 5^th^ intron constructs (N–Q). Constructs were bombarded into four independent floral buds, and results are representative of three independent bombardment experiments. Scale bar  =  0.5 mm.

The longest introns of *AG* and *FLC* in *Arabidopsis* play a regulatory role for their gene expression [12], [13]. It was intriguing to know whether the introns of *PeMADS4* may regulate its distinct expression at the late floral organ primordia stage of *Phalaenopsis* orchids. To test this, we first sequenced the genomic sequence of BAC clones containing *PeMADS4*, NCKU-PE-btBAC-1105 H24. Then, the genomic sequence was compared to its cDNA sequence, and seven exons and six introns were identified for *PeMADS4* with a long 5^th^ intron of 9,483 bp (Fig. 6A). The 5^th^ intron contains two conserved CC(A/T)_6_GG motifs (Fig. 6A, rhombus) and 11 C(A/T)_8_G motifs (Fig. 6A, triangles) (Fig. 6A).

To assess the effect of the 5^th^ intron on *PeMADS4* expression, we generated three subfragments of 8-kb, 3.5-kb, and 2-kb fragments by PCR amplification, cloned into the upstream region of the promoter sequence in the pBI-Pe4p-375 construct, and named as pBI-Pe4p-375+8 kb, pBI-Pe4p-375+3.5 kb, and pBI-Pe4p-375+2 kb 5^th^ intron constructs, respectively (Fig. 6A). The addition of the 8-kb fragment intron resulted a sharp decrease of GUS expression in all four floral organs (Fig. 6F–I) as compared with the pBI-Pe4p-375 native construct (Fig. 6B–E), which suggests that the 8-kb 5^th^ intron may have a negative effect on *PeMADS4* expression. Alternatively, the addition of the 8-kb fragment was too long to affect the transformation efficiency. Otherwise, the GUS expression in all floral organs was not significantly different with the addition of either the 2- or 3.5-kb fragments of the 5^th^ intron (Fig. 6J–Q), so the 3.5- or 2-kb 5^th^-intron sequence showed little or no effects for the exclusive *PeMADS4* expression in lip and column.

### DNA methylation was not responsible for regulation of *PeMADS4* expression

**Figure 7:**
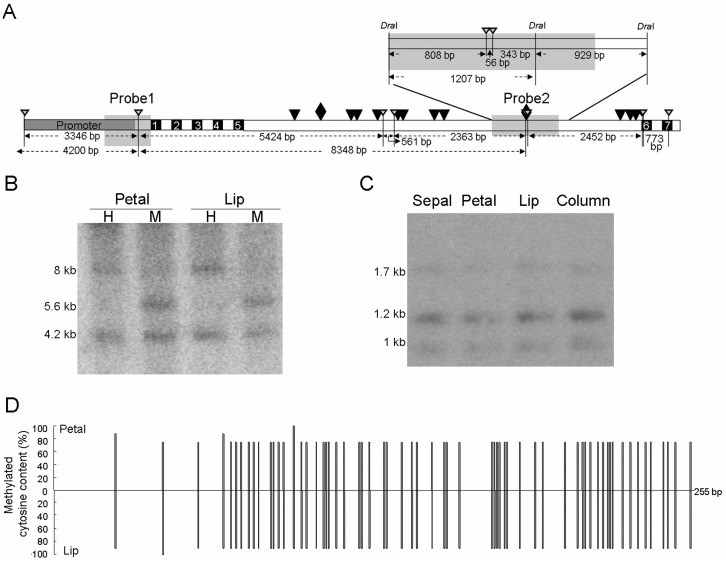
Methylation status in the promoter and 5^th^ intron regions of *PeMADS4*. (A) Locations of the probes used for Southern blot analysis and methylation status in the promoter (B) and 5^th^ intron (C) regions of *PeMADS4*. Gray, black, and white boxes indicate the promoter, exon, and intron regions of *PeMADS4* gene, respectively. The rhombus and black triangles are the predicted CArG boxes. The white triangles point to the *Hpa*II/*Msp*I sites. Probes used in this study are shown in gray located in the 5′ UTR (Probe 1) or in the 5^th^ intron (Probe 2). (B) Southern blot analysis was performed with genomic DNA extracted from petal and lip of *P. equestris*, digested with methylation-sensitive enzymes, *Hpa*II (H) and *Msp*I (M), and hybridized with probe 1. (C) Southern blot analysis was performed with the genomic DNA extracted from sepals, petals, lips and columns of *P. equestris*, double-digested with *Dra*I and *Hpa*II, and hybridized with probe 2. Probe 2 was a 2,136-bp fragment between three *Dra*I restriction enzyme cleavage sites and contained two *Hpa*II site. (D) Bisultife sequencing for the methylation status within the promoter region of *PeMADS4*.

To examine whether the specific expression of *PeMADS4* in lip and column was caused by DNA methylation in the regulatory sequences *in planta*, Southern blot hybridization was performed to analyze the DNA methylation status in the promoter, translation start site, and intron regions of *PeMADS4*. DNA samples isolated from petal and lip of *P. equestris* were digested with the methylation-sensitive endonucleases *Hpa*II or *Msp*I (H or M) (Fig. 7B) and *Dra*I/*Hpa*II (Fig. 7C). Probe 1 containing a 582-bp fragment including a 375-bp promoter sequence and a 207-bp 5′-UTR region of *PeMADS4* (Fig. 7A, B) was used for DNA samples from petal or lip and similar methylation status were obtained with digestion of *Hpa*II (Fig. 7B, 4.2- and 8-kb fragments) and *Msp*I (Fig. 7B, 4.2- and 5.6-kb fragments). Moreover, hybridization with probe 2, containing a 2,136-bp fragment of the 5^th^ intron of *PeMADS4*, gave the same results for all *Dra*I/*Hpa*II-digested DNA samples from sepal, petal, lip, and column (Fig. 7A, C, 1- and 1.2-kb fragments), so the *Hpa*II site within probe 2 region was methylated in all four floral organs. Thus, the DNA methylation status was the same in the promoter region, translation start site, and the 5^th^ intron regions of the *PeMADS4* gene for both petal and lip of *Phalaenopsis* flowers.

To have a single-base resolution of methylation status, bisulfite sequencing technology was performed. Highly methylated cytosine residues were detected within the promoter region and translation start site of *PeMADS4* in DNA samples from both petal and lip (Fig. 7D). Therefore, DNA methylation may not play a role if any in tissue specificity of *PeMADS4* expression in lip and column.

### Concomitant differential histone acetylation for *PeMADS4* expression

**Figure 8:**
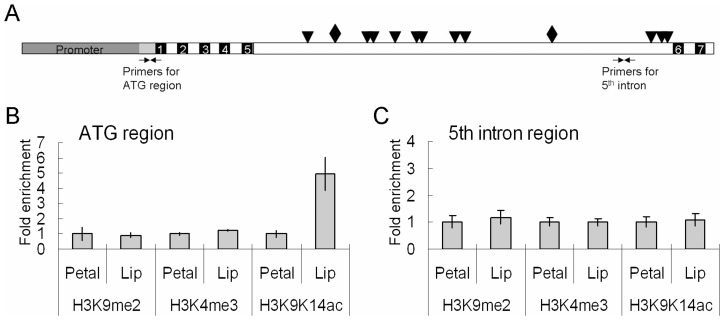
Histone modification on the ATG and 5^th^ intron regions of *PeMADS4*. (A) Locations of the primers used in ChIP assay. Gray, black, and white boxes indicate the promoter, exon, and intron regions of *PeMADS4* gene, respectively. The rhombus and black triangles are the predicted CArG boxes. ChIP assay of histone modification of dimethyl-H3K9 (H3K9me2), trimethyl-H3K4 (H3K4me3), acetyl-H3K9 and H3K14 (H3Ac) was analyzed on the ATG (B) and 5^th^ intron regions (C) of *PeMADS4* in the petal and lip of *P. equestris*. The amount of DNA after ChIP was quantified and normalized to an internal control *ACTIN2* for H3K4me3 and H3K9K14ac or *Ta3* for H3K9me2. Data are mean ± SD calculated from three technological and two biological replicates. X =  fold.

It is possible that the tissue-specific expression profiles of *PeMADS* genes not only reside in the DNA-level promoter sequences, but also in the protein-level histone modification. To address this, various histone modifications were analyzed by ChIP assay with antibodies against the gene repression marker H3K9me2 and gene activation markers H3K4me3 and H3K9K14ac. The precipitated DNA samples from both petal and lip were analyzed by real-time PCR with the primer sequences located at the translation start site (ATG) and the 5^th^ intron regions of *PeMADS4* (Fig. 8A). Notably, we detected a 4.9-fold higher H3K9K14ac at the translation start site in lip than in petal (Fig. 8B). In contrast, no differential levels of H3K9me2 and H3K4me3 were detected in the translation start site in both petal and lip (Fig. 8B). Furthermore, no substantial differential levels of H3K9me2, H3K4me3, and H3K9K14ac within the 5^th^ intron region of *PeMADS4* were detected in petal and lip (Fig. 8C). Thus, the increased level of H3K9K14ac on the translation start site of *PeMADS4* gene may allow for more access of the transcription factor and the further increased gene expression in lip, thus leading to its optimized expression in *Phalaenopsis*.

## Discussion

In model species *Arabidopsis thaliana* and *Antirrhinum majus*, the molecular genetic studies on flower morphogenesis indicate that homeotic B-class MADS-box genes determine petals and stamen identities. A shift model has been proposed through several comparative expression analyses suggesting that a shift expression of B-class genes to the outer perianth is associated with the petal-like organs on the first flower whorl in several monocot species, such as *Tulipa gesneriana* and *Lilium longiflorum* [44], [45]. To understand the development and evolution of orchid flowers, extensive studies of the molecular phylogeny and expression patterns of candidate B-class MADS-box genes have been performed in several genus, species, and hybrids of orchids. Four ancient orchid-specific clades of *AP3*-like and one major lineage of *PI*-like B-class genes were identified and characterized [17], [26], [46]. Differential expression of *AP3*-like genes has been associated with the identity of distinct perianth organs, that are the basis of several models used to explain the morphogenesis and evolution of the orchid flowers [20], [27].

### Histone acetylation and promoter sequences act synergistically to regulate *PeMADS4* expression in lip

The minimum promoter sequence necessary for a wild-type *AP3* expression pattern in *Arabidopsis* is localized within the 727-bp fragment upstream of the transcriptional start site [9]. In this study, we showed that the minimal promoter sequences for *PeMADS2∼6* were 291 bp, 407 bp, 375 bp, 122 bp, and 208 bp of their upstream regulatory sequences, respectively. In addition, the regulation of the promoter sequence and the increased H3K9K14ac level may act synergistically to result in the exclusive high expression of *PeMADS4* in lip and column at the late floral organ primordia stage and floral bud stage of *Phalaenopsis* orchids. A similar regulatory mechanism may be adopted for regulation of the distinct expression profiles of the other three *AP3*-like paralogs, *PeMADS2*, *PeMADS3*, and *PeMADS5*, in *Phalaenopsis* flowers to complete the diversified subfunctionalization for orchid floral morphogenesis.

### Transient expression assay for promoter analysis by use of particle bombardment

Stable transformation of *Phalaenopsis* orchids is time-consuming and requires considerable human resources, because their long life cycles of two to three years for the transition from the seed germinative to reproductive stages. Transient expression assays, which were carried out by using particle bombardment [47], [48] and protoplast transfection [49], have been used to reduce the time for analyzing gene functions in *Arabidopsis* [50], [51], rice [49], [52], maize [53], potato [54], soybean [55], tomato [56], wheat [55], and white spruce [57]. Protoplasts retain many signal transduction pathways from the cells which they are derived [58], but the process of protoplast cultivation may change the mRNA expression profiles [59]. In contrast, particle bombardment permits the transient expression within intact tissues of entire plants. However, an important argument for transient expression is that it is easy to overexpress gene constructs because of high copy numbers of plasmid DNA, strong promoters, long expression times, and the independence of the transgene expression on the genomic site of integration [60]. While in stable transgenic plants, a high transgene copy is frequently accompanied with gene silencing and the position effect affects the expression of promoter constructs by flanking sequences in the genome. In Orchidaceae, particle bombardment with histochemical GUS staining have been used to analyze the promoter activities of *disease resistance response protein* (*OnDRRP*), *Expansin* (*OnExpansin*), and three *trypsin inhibitor* (*OnTI1∼3*) in leaves and flowers of *Oncidium* Gower Ramsey, and *cytokinin oxidase* (*DSCKX1*) in protocorm-like-bodies of *Dendrobium* Sonia, respectively [61], [62]. Here, we analyzed the promoter activities of *PeMADS2*∼*6* for driving GUS and luciferase reporter genes by particle bombardment, and the ubiquitous expression in all floral organs may be caused by the high copies of bombarded plasmid DNA and/or the naked DNA lack of chromatin modification. However, the serial deletion sequences of *PeMADS4* promoter showed a higher luciferase activity in lip and column than in sepal and petal in contrast to the more-or-less similar lucifease activity detected in all four floral organs driven by *PeMADS6* promoter. Moreover, we further examined the DNA methylation and histone modification within the translation start site of *PeMADS4* to verify the regulatory strategy for its differential expression pattern. All these results suggested that the transient expression assay by particle bombardment accompanying with DNA methylation and histone modification analyses provides a basic information about the regulatory strategies of these *PeMADS2∼6* genes exhibiting distinct expression profiles.

### 375-bp promoter sequence of *PeMADS4* was required for its lip and column expression

The 375-bp promoter sequence of *PeMADS4* conferred higher luciferase activity in lip and column, which meant that the *P*e*MADS4* promoter was regulated by a lip- and column-specific transcription factor. Several transcription factors have been shown to dominantly express in lip, such as MADS, ARF, C3H, HB-other, YABBY, ZF-HD, bZIP, CO, TALE, HD-ZIP, MYB, and AP2-like families [63]. The *cis*-acting regulatory elements on the upstream region of *PeMADS4* were analyzed by PLACE software, and the CARGCW8GAT for MADS and MYBCOREATCYCB1 or MYBST1 for MYB-binding motifs were predicted within this sequence (S2 Table). It is possible that unidentified motifs were resided in the 375-bp fragment of *PeMADS4* promoter for the interaction with above mentioned transcription factors. The exact transcription factors responding for the activation on the 375-bp fragment of *PeMADS4* promoter were required for further studies.

### The promoter sequences of *OrcPI* and *PeMADS6* showed little similarity

Comparing the differential expression patterns of *AP3*-like genes, *PI*-like genes showed uniformly and highly conserved expression patterns in all four floral organs in several species of various subfamily of Orchidoideae, such as Cypripedioideae, Epidendroideae, Orchidoideae, and Vanilloideae [20]. *OrcPI* is a *PI*-like MADS gene from *Orchis italica*, a species of Orchidoideae, and expresses in young inflorescences and all floral organs [42]. Various serial deletion of promoter sequence of *OrcPI* with 1324-bp, 854-bp, 577-bp, and 356-bp upstream sequences can drive the GUS expression in petal tissue in the white *Rosa hybrid* [42]. However, the upstream regulatory sequences of *PeMADS6* and *OrcPI* could not be aligned together, even for their minimal promoter with 208- and 356-bp fragments, respectively. However, it is intriguing that two 11-bp motifs were detected between nucleotide −249 and −173 bp of *PeMADS6* promoter and between −937 and −641 bp of *OrcPI* promoter by using the BLAST2 algorithm (S4 Figure). Whether this element plays any roles in the ubiquitous expression of the PI-like genes in orchid flowers awaits further studies.

### Histone modification regulated the plant development and stress response

The epigenetic regulation is dynamic and varies between cell types and in response to development stages or environmental stimuli [64]. H3K9me2 is mainly detected in heterochromatin regions and associated with transposable elements (TEs) [65]. In contrast, H3K4me3 is enriched in euchromatic regions and associated with transcribed regions of non-TE genes. Expectedly, typical activating histone modification, such as H3K4me3 and H3K9ac is detected in the same genomic regions [66]. For example, during the vernalization response (exposure to a prolonged period of low temperature), the gene repression marks, H3K9me2 and H3K27me2, are enriched at *FLC* locus and thereby controlling flowering time, in contrast to the activated state of *FLC* chromatin with active histone marks, H3K4me3 and H3ac, before prolonged cold exposure [67]. Moreover, several epigenetic regulators are involved in the regulation of floral homeotic genes. Mutation in a H3K4 methyltransferase *ATX1* results down-regulation of *AP1*, *AP2*, *PI*, and *AG*, but not of *AP3* and *SEP3* [68]. The PRC2-like complexes containing CLF, FIE, EMF2 and MSI1 act on repression of *AG* by regulating H3K27me3 [69], [70], and the *emf2* mutant results ectopic overexpression of *AG*, *AP3*, *AP1*, *PI*, *SEP2*, and *SEP3* [71]. Furthermore, the gene repression marker, H3K27me3, have been shown that its release results in tissue-specific gene activation [72]. Here, we showed that an increased level of H3K9K14ac on the translation start site of *PeMADS4* gene may enhance the exclusive gene expression in lip to decipher the lip morphogenesis in *Phalaenopsis* orchids, although its mechanism is still needed to be investigated.

### Transgenic plants by use of the *PeMADS2∼5* promoter

In *Arabidopsis*, the promoter sequences of *AP3* and *PI* have been used for the floral organ-specific expression in the transgenic approach. The 288-bp promoter sequence of *AP3* with a petal-specific domain [9] and the 300-bp fragment of *PI* with expression in petal and stamen [11] were used to drive an RNAi vector targeting the GUS reporter gene and introduced into a line constitutively expressing GUS, which resulted in reduced GUS expression in petal [73]. However, the promoter sequences of *PeMADS2*∼*5* drove the expression of GUS and luciferase reporter genes in the whole flower but not exclusively in distinct floral organs. Thus, the amplified upstream regulatory sequences of *PeMADS2*∼*5* could be used as flower-specific, but not practically for floral organ-specific promoters in the application for transgenic plants.

## Supporting Information

S1 Figure **Functional analysis of serial deletions of** ***PeMADS2*** **promoter.** (A) Serial deletion constructs of *PeMADS2* promoter. (B–Q) Histochemical assay of flower organs bombarded with serial deletions of *PeMADS2* promoter shown in the order of pBI-Pe2p-2224 (B-E), pBI-Pe2p-1823 (F-I), pBI-Pe2p-1312 (J-M), pBI-Pe2p-750 (N–Q), and pBI-Pe2p-291 (R–U). Constructs were bombarded into four independent floral buds, and results are representative of three independent bombardment experiments. Scale bar  =  0.5 mm.
(TIF)

S2 Figure **Functional analysis of serial deletions of** ***PeMADS3*** **promoter.** (A) Serial deletion constructs of *PeMADS3* promoter. (B–Q) Histochemical assay of flower organs bombarded with serial deletions of *PeMADS3* promoter shown in the order of pBI-Pe3p-1007 (B–E) and pBI-Pe3p-407 (F–I). Constructs were bombarded into four independent floral buds, and results are representative of three independent bombardment experiments. Scale bar  =  0.5 mm.
(TIF)

S3 Figure **Functional analysis of serial deletions of** ***PeMADS5*** **promoter.** (A) Serial deletion constructs of *PeMADS5* promoter. (B–Q) Histochemical assay of flower organs bombarded with serial deletions of *PeMADS5* promoter shown in the order of pBI-Pe5p-1507 (B-E), pBI-Pe5p-1053 (F–I), pBI-Pe5p-441 (J–M), and pBI-Pe5p-122 (N–Q). Constructs were bombarded into four independent floral buds, and results are representative of three independent bombardment experiments. Scale bar  =  0.5 mm.
(TIF)

S4 Figure **Alignment of the promoter sequences of** ***PeMADS6*** **and** ***OncPI*** **.**
(TIF)

S1 Table **Primers used in this study.**
(DOC)

S2 Table ***Cis*** **-acting regulatory elements on the upstream region of** ***PeMADS4*** **.**
(DOC)
