# Supplementary figures and images for: Histone Acetylation Accompanied with Promoter Sequences Displaying Differential Expression Profiles of B-Class MADS-Box Genes for Phalaenopsis Floral Morphogenesis
Source: PLoS One. 2014 Dec 11;9(12):e106033. doi: 10.1371/journal.pone.0106033 (PMC4263434; doi:10.1371/journal.pone.0106033)

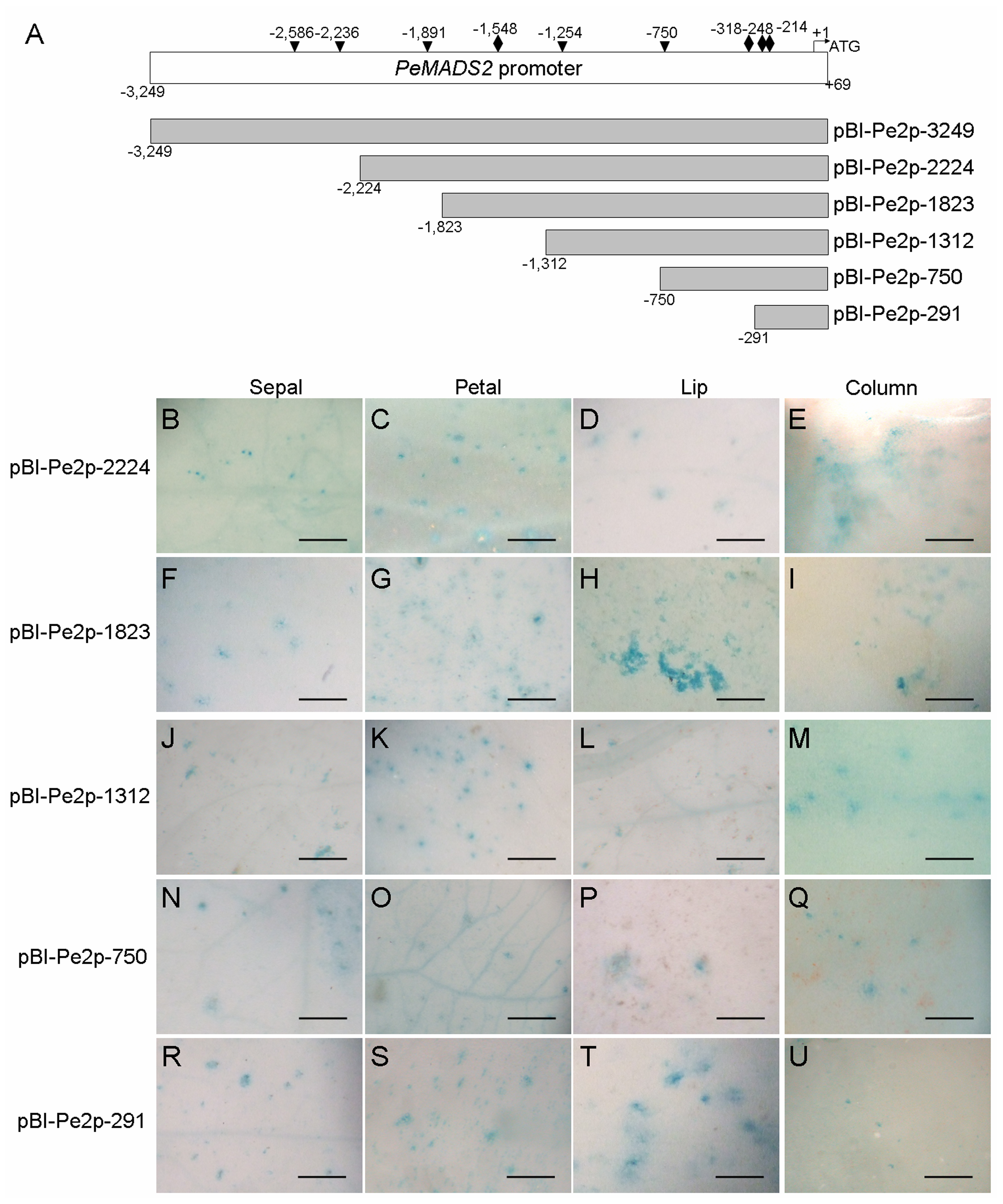

Supplement: S1 Figure — Functional analysis of serial deletions of PeMADS2 promoter. (A) Serial deletion constructs of PeMADS2 promoter. (B–Q) Histochemical assay of flower organs bombarded with serial deletions of PeMADS2 promoter shown in the order of pBI-Pe2p-2224 (B-E), pBI-Pe2p-1823 (F-I), pBI-Pe2p-1312 (J-M), pBI-Pe2p-750 (N–Q), and pBI-Pe2p-291 (R–U). Constructs were bombarded into four independent floral buds, and results are representative of three independent bombardment experiments. Scale bar = 0.5 mm. (TIF) [file pone.0106033.s001.tif]

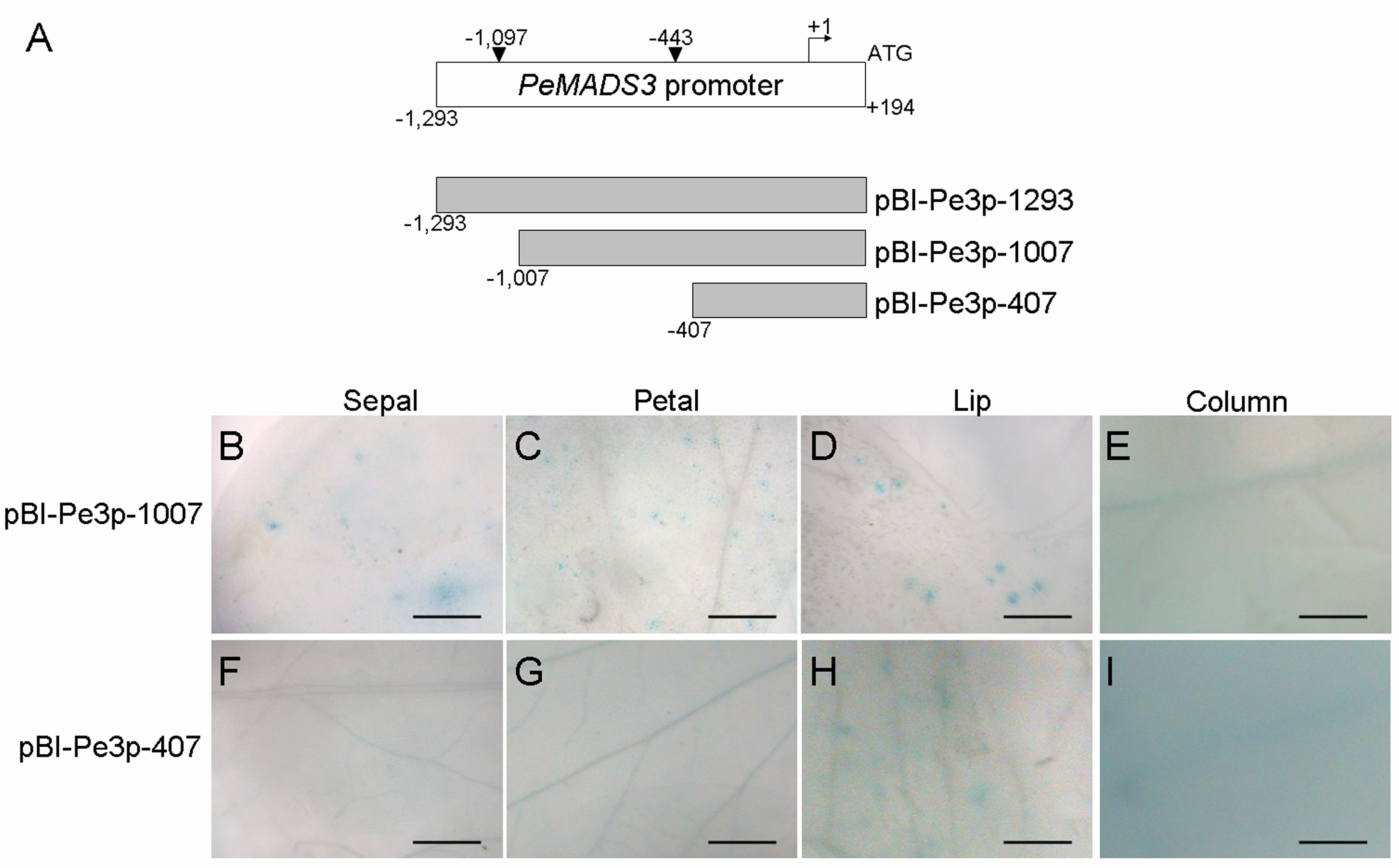

Supplement: S2 Figure — Functional analysis of serial deletions of PeMADS3 promoter. (A) Serial deletion constructs of PeMADS3 promoter. (B–Q) Histochemical assay of flower organs bombarded with serial deletions of PeMADS3 promoter shown in the order of pBI-Pe3p-1007 (B–E) and pBI-Pe3p-407 (F–I). Constructs were bombarded into four independent floral buds, and results are representative of three independent bombardment experiments. Scale bar = 0.5 mm. (TIF) [file pone.0106033.s002.tif]

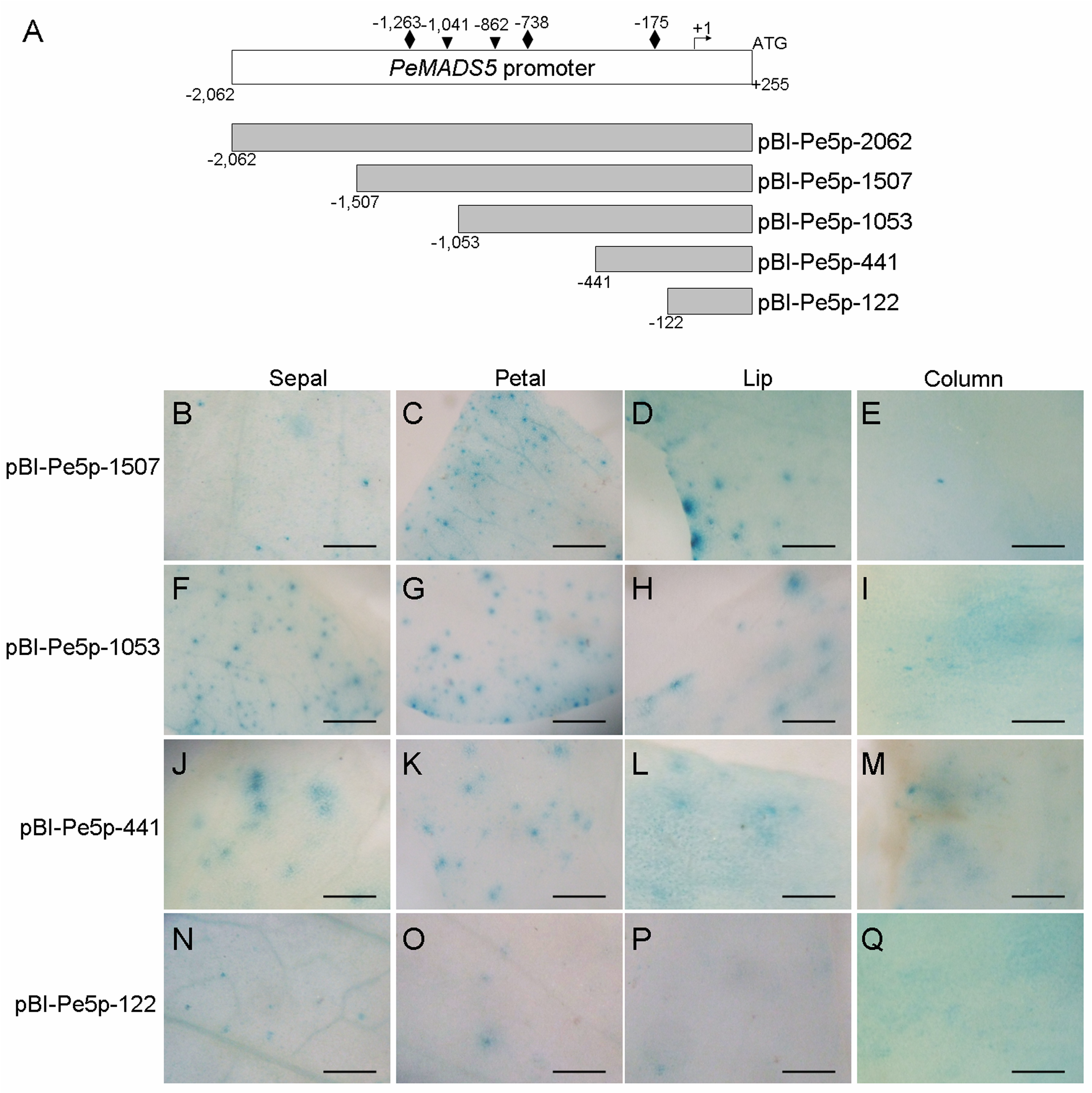

Supplement: S3 Figure — Functional analysis of serial deletions of PeMADS5 promoter. (A) Serial deletion constructs of PeMADS5 promoter. (B–Q) Histochemical assay of flower organs bombarded with serial deletions of PeMADS5 promoter shown in the order of pBI-Pe5p-1507 (B-E), pBI-Pe5p-1053 (F–I), pBI-Pe5p-441 (J–M), and pBI-Pe5p-122 (N–Q). Constructs were bombarded into four independent floral buds, and results are representative of three independent bombardment experiments. Scale bar = 0.5 mm. (TIF) [file pone.0106033.s003.tif]

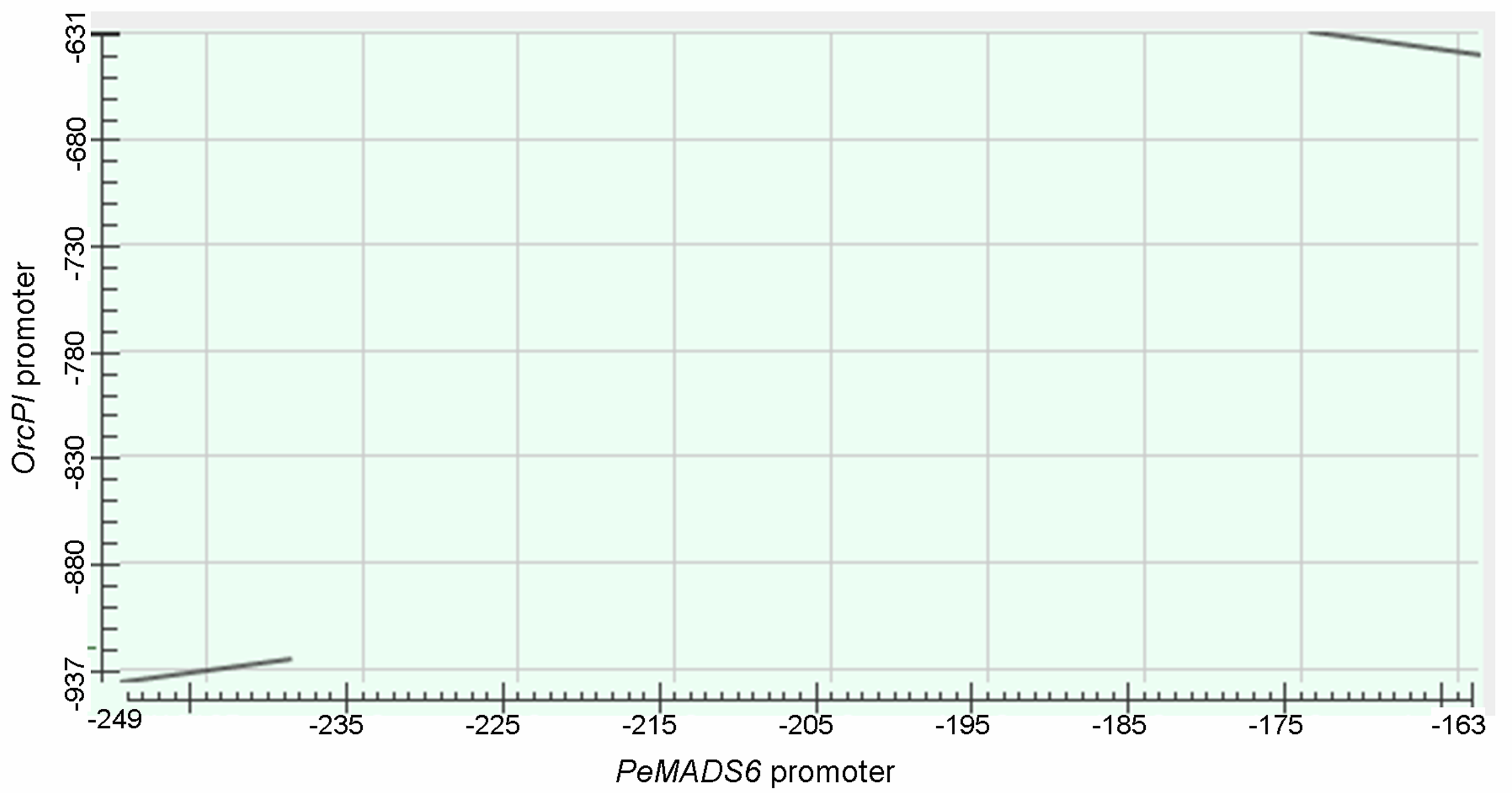

Supplement: S4 Figure — Alignment of the promoter sequences of PeMADS6 and OncPI . (TIF) [file pone.0106033.s004.tif]
