# Supplementary material for: Histone Acetylation Accompanied with Promoter Sequences Displaying Differential Expression Profiles of B-Class MADS-Box Genes for Phalaenopsis Floral Morphogenesis
Source: PLoS One. 2014 Dec 11;9(12):e106033. doi: 10.1371/journal.pone.0106033 (PMC4263434; doi:10.1371/journal.pone.0106033)
Supplement: S1 Table — Primers used in this study. (DOC) [file pone.0106033.s005.doc]

**Table S1.** Primers used in this study.

| Primer name | Sequence (5’-3’) |
| --- | --- |
| **Serial deletion of *PeMADS2~6* promoter constructs** | |
| Pe2pF3249SphI | GCATGCCCTTCCTCGCAGGTGTGTCC |
| Pe2pF2224SphI | GCATGCGGCACGACTCTAAAACTCACCAAG |
| Pe2pF1823SphI | GCATGCTACCTATAAGCTACGTGAAAACAATACAT |
| Pe2pF1312SphI | GCATGCCAGTACAATCTGAATCAGGAAACCCTATT |
| Pe2pF750SphI | GCATGCCACTCTCTATATTAAGGGGAAAGGGAAAC |
| Pe2pF291SphI | GCATGCTTATCTCTGCCCGCAACTCCTTT |
| Pe2pR1BamHI | GGATCCCTCTCTCTTCCCCTGTTCTCCCC |
| Pe3pF1293PstI | CTGCAGGATACTACGTTAAAAGAACTAGGAATC |
| Pe3pF1007PstI | CTGCAGCCTACCCTCACCCAATCC |
| Pe3pF407PstI | CTGCAGTATTAAGGAAAATTGAATGGTAGTTTG |
| Pe3pR1XbaI | TCTAGAGGTTCTCTTTCTTGCTCTGCTTC |
| Pe4pF3303SphI | GCATGCGAATGCCCACAGGCCG |
| Pe4pF2313SphI | GCATGCCGAGAGAGGCGATTGTGGAGG |
| Pe4pF1497SphI | GCATGCGGACCGTCGAAGCACCTACC |
| Pe4pF935SphI | GCATGCCCACTCTCAGGATTCGACCACTCTC |
| Pe4pF375SphI | GCATGCGAAGTATTCCACTTTGTTCCCCATTATTT |
| Pe4pR1BamHI | GGATCCCTTCAAATCTCTTTTCTCCACTCC |
| Pe5pF2062PstI | CTGCAGGGCGAGCTTGTTAGTATAAAG |
| Pe5pF1507PstI | CTG CAGATTTGAATCGGTCACCTTG |
| Pe5pF1053PstI | CTGCAGCTTGAGATTCATCTTTATATTGC |
| Pe5pF441PstI | CTGCAGTTTGAGCTTTAGCTTGGGTTAAGGTG |
| Pe5pF122PstI | CTGCAGCTTGTGTTTGGGCTAGGTTAGGCA |
| Pe5pR1XbaI | TCTAGATCCCTCCACCCCCCTTAGC |
| Pe6pF1514PstI | CTGCAGTTGCTTCCACCTTTGTTTGTT |
| Pe6pF1108PstI | CTGCAGGTTGCCTTAACAACTATTCTCTC |
| Pe6pF808PstI | CTGCAGGATAAATAAGACTTACACCCTTAG |
| Pe6pF508PstI | CTGCAGGAACTCATGAGAGGGAAATATGA |
| Pe6pF208PstI | CTGCAGACTGAGGGGCTGAGTGAAAAA |
| Pe6p-R1XbaI | CCTCTAGACATCTCCACCCAAAAACCGG |
| **Serial deletion of 5^th^ intron of *PeMADS4*** | |
| P4-5inRevSphI | GCATGCCTGCAACAAATGAAGAAGAGGGGTAAG |
| P4-5in1.5kbForSphI | GCATGCCCACATTTTCGTCACTCTCTCCTTTCTTATG |
| P4-5in3kbForSphI | GCATGCCCAAGAGACTCCAAATCCTGGTATTATGAGTC |
| P4-5in8kbForSphI | GCATGCGCTTTTGAGACTTTGGTTTCTCCCC |
| **Southern blot probes, bisulfite sequencing, and ChIP assay** | |
| Pe4pProbe1F | CTATCGCTTTTGCGTTTCCAGTTC |
| Pe4pProbe1R | TCCCTCGCCTTCTTCATAATCCC |
| Pe4-5intProbe2F | GTCGATTCGTACAGGTGCTTTATATTATTGG |
| Pe4-5intProbe2R | CGAGATAAAGGCTTGTTATTGTTGTTGTTG |
