# Supplementary material for: Histone Acetylation Accompanied with Promoter Sequences Displaying Differential Expression Profiles of B-Class MADS-Box Genes for Phalaenopsis Floral Morphogenesis
Source: PLoS One. 2014 Dec 11;9(12):e106033. doi: 10.1371/journal.pone.0106033 (PMC4263434; doi:10.1371/journal.pone.0106033)
Supplement: S2 Table — Cis -acting regulatory elements on the upstream region of PeMADS4 . (DOC) [file pone.0106033.s006.doc]

**Table S2.** *Cis*-acting regulatory elements on the upstream region of *PeMADS4*.

| Factor or site name | Location (current or opposite strand) | Signal sequence |
| --- | --- | --- |
| ARR1AT | -37(+)  82(-)  150(-)  197(+) | NGATT |
| ASF1MOTIFCAMV | 137(-) | TGACG |
| BIHD1OS | -49(-) | TGTCA |
| BOXIINTPATPB | 70(-) | ATAGAA |
| CAATBOX1 | -285(-)  26(+) | CAAT |
| CACTFTPPCA1 | -373(-)  -366(+)  -296(+)  -123(-)  -94(+)  -67(+)  0(+)  9(+)  146(+)  153(+)  165(-)  185(-) | YACT |
| CARGCW8GAT | -65(+) | CWWWWWWWWG |
| CATATGGMSAUR | -224(+) | CATATG |
| CGCGBOXAT | -13(+)  -11(+) | VCGCGB |
| DOFCOREZM | -364(-)  -125(+)  -59(+)  11(-)  55(-)  77(+)  96(-)  114(-)  193(+) | AAAG |
| DPBFCOREDCDC3 | -20(+) | ACACNNG |
| E2FCONSENSUS | 22(-) | WTTSSCSS |
| EBOXBNNAPA | -224(+) | CANNTG |
| GATABOX | -329(-)  -261(-)  -232(-)  -212(-)  -194(-)  -188(-)  -150(-)  -144(-)  50(-) | GATA |
| GT1CONSENSUS | -331(-)  38(-)  97(-) | GRWAAW |
| GT1GMSCAM4 | 97(-) | GAAAAA |
| GTGANTG10 | -322(-)  -292(-)  -42(+)  8(-)  145(-)  152(-) | GTGA |
| IBOXCORE | -330(-) | GATAA |
| INRNTPSADB | -287(-)  7(+) | YTCANTYY |
| MARTBOX | -315(-)  -313(-) | TTWTWTTWTT |
| MYBCOREATCYCB1 | 132(-) | AACGG |
| MYBST1 | -329(-) | GGATA |
| MYCCONSENSUSAT | -224(+) | CANNTG |
| NODCON2GM | 194(-) | CTCTT |
| NTBBF1ARROLB | -126(-) | ACTTTA |
| OSE2ROOTNODULE | 194(-) | CTCTT |
| POLASIG1 | -310(+) | AATAAA |
| POLASIG3 | -353(-) | AATAAT |
| POLLEN1LELAT52 | 99(-)  115(-)  190(+) | AGAAA |
| PYRIMIDINEBOXOSRAMY1A | 76(-) | CCTTTT |
| RAV1AAT | -339(+)  -22(+)  140(+) | CAACA |
| ROOTMOTIFTAPOX1 | -273(-)  -244(-) | ATATT |
| SEF4MOTIFGM7S | -336(-) | RTTTTTR |
| SORLREP3AT | -240(+)  -220(+)  -164(+)  -91(+) | TGTATATAT |
| SREATMSD | -330(+) | TTATCC |
| TAAAGSTKST1 | -126(+) | TAAAG |
| TATABOX4 | -266(-)  -249(+)  -130(+)  -121(+)  -72(-) | TATATAA |
| TATABOX5 | -352(+)  -311(-) | TTATTT |
| TATABOXOSPAL | -351(+) | TATTTAA |
| TATAPVTRNALEU | -130(-) | TTTATATA |
| TBOXATGAPB | -365(+) | ACTTTG |
| WBOXATNPR1 | 138(-) | TTGAC |
| WRKY71OS | -49(+)  138(-) | TGAC |
